# Recurrent Paraspinal Abscess and Persistent Bacteremia Complicated by Mitral Valve Endocarditis and Septic Embolic Stroke

**DOI:** 10.7759/cureus.108339

**Published:** 2026-05-06

**Authors:** Duong Huynh, Kamyar Chong, Joshua Can, Shriya Patel, Khiet T Nguyen, Abeera Rabbani, Anubhav Poudel, Roxana Lazarescu

**Affiliations:** 1 Internal Medicine, Wyckoff Heights Medical Center, Brooklyn, USA; 2 Internal Medicine, Touro College of Osteopathic Medicine (TouroCOM) Harlem Campus, New York, USA; 3 Internal Medicine, Saba University School of Medicine, The Bottom, BES; 4 Internal Medicine, Xavier University School of Medicine, Oranjestad, ABW; 5 Medicine, Interfaith Medical Center, Brooklyn, USA

**Keywords:** end-stage renal disease (esrd), hemodialysis, infective endocarditis, paraspinal abscess, polymicrobial bacteremia, septic emboli, vegetation

## Abstract

Patients with end-stage renal disease (ESRD) on hemodialysis are at high risk for infective endocarditis (IE), recurrent bacteremia, and embolic complications, with in-hospital, six-month, and one-year mortality rates exceeding those of the general population. This case involves a 62-year-old man with ESRD on hemodialysis who has a history of lumbar spinal fusion with hardware and a prior paraspinal abscess, who presented with septic shock and diffuse pain in the setting of recurrent polymicrobial multidrug-resistant bacteremia. The patient’s hospital course was complicated by mitral valve IE and multifocal septic embolic strokes. Despite broad-spectrum antimicrobial therapy and percutaneous drainage of the abscess, source control was not achieved in this patient due to the inability to undergo surgical intervention. Cardiothoracic surgery recommended abscess control prior to mitral valve replacement; however, neurosurgery deemed him prohibitively high risk due to severe thrombocytopenia and persistent hypotension. With ongoing bacteremia and clinical deterioration, goals-of-care discussions resulted in DNR/DNI status and transition to comfort measures. This case underscores the unfavorable prognosis that IE carries in chronic hemodialysis patients and emphasizes the need for early diagnosis, aggressive efforts at source control, and timely multidisciplinary assessment for overall care and definitive surgical intervention.

## Introduction

Chronic hemodialysis substantially elevates the risk of infective endocarditis (IE), with an approximately 18-fold higher incidence compared to non-dialysis populations, driven by frequent vascular instrumentation, biofilm formation on access sites, and underlying immune compromise [1,2]. Outcomes for dialysis patients with IE have markedly higher rates of systemic embolization, heart failure, and in-hospital mortality, which have been reported as high as 20-50% [1-7]. Left-sided valvular involvement in chronic hemodialysis patients can cause complications such as systemic embolization and can increase in-hospital mortality up to 40% [2-7].

Septic emboli complicate a substantial proportion of IE cases, particularly when mitral vegetations exceed 10 mm or when a *Staphylococcus aureus* infection is implicated [6,8,9]. Hemodialysis patients face a variety of challenges, including multidrug-resistant pathogens, unstable drug clearance, and ineligibility for valve surgery [2-5,7,10,11]. Specifically, treatment options in this population are constrained by limited antibiotic dosing options due to nephrotoxicity concerns and significantly reduced surgical candidacy, with cardiac surgery rates reported as low as 6-10% among hemodialysis patients with IE [1,2,8]. Although gram-positive cocci remain the predominant cause of spinal hardware infections, gram-negative and polymicrobial infections are involved in a minority of cases and are frequently associated with more resistant pathogens and worse clinical outcomes [12].

Septic embolic stroke occurs when fragments of infected vegetation composed of bacteria, fibrin, platelets, and inflammatory cells detach from a cardiac valve, travel through the systemic arterial circulation, and occlude cerebral arteries, causing ischemic infarction [6]. In left-sided IE, vegetations on the mitral or aortic valve are directly exposed to the high-pressure systemic circulation, and when these friable masses fragment, embolic debris is ejected into the aorta and preferentially lodges in the cerebral vasculature [6].

This case report describes a 62-year-old male with end-stage renal disease (ESRD) on hemodialysis with prior lumbar spinal fusion who developed mitral IE and multifocal septic embolic strokes secondary to uncontrolled paraspinal infection and recurrent bacteremia. This case highlights how persistent deep foci of infection can prevent source control, limit therapeutic options, and dramatically increase embolic and mortality risk in an already vulnerable population.

## Case presentation

A 62-year-old male with ESRD on hemodialysis via a left internal jugular tunneled dialysis catheter presented to the emergency department with generalized body pain and weakness. His past medical history included nonobstructive coronary artery disease, hypothyroidism, a lumbar fusion of L5-S1 20 years ago, concurrent methicillin-resistant *S. aureus *and *Stenotrophomonas maltophilia *bacteremia two years prior secondary to an abscess adjacent to the right internal jugular PermCath, discitis-osteomyelitis of L4-L5 with *Enterococcus gallinarum* bacteremia six months prior treated with six weeks of IV antibiotics, and recurrent discitis-osteomyelitis of L4-L5 with an associated left paraspinal abscess that was percutaneously drained and treated with six weeks of IV antibiotics three months before presentation. The patient was not on suppressive antibiotic therapy at the time of presentation.

**Figure 1:**
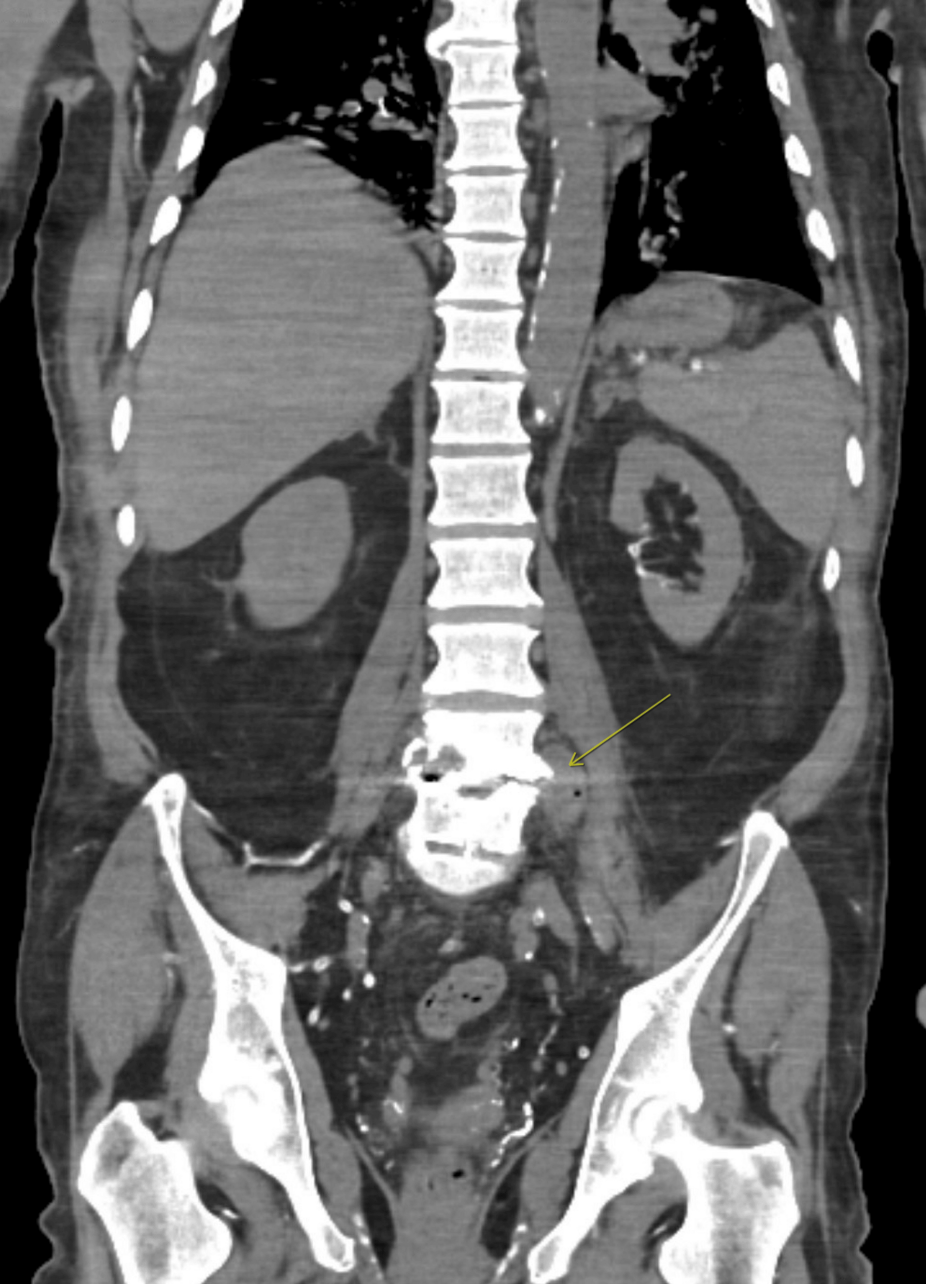
Coronal CT of the abdomen and pelvis without IV contrast demonstrating a left paraspinal abscess (yellow arrow)

On arrival, the patient had severe hypotension with a blood pressure of 60/40 mmHg but remained alert and oriented, endorsing diffuse body pain and weakness. Cardiac auscultation revealed no appreciable murmurs. Leukocytosis (19 × 10⁹/L), thrombocytopenia (platelets 60 × 10⁹/L), metabolic acidosis (pH 7.18, bicarbonate 9 mmol/L), elevated transaminases, and elevated troponin consistent with type II myocardial injury were seen on laboratory studies (Table 1). CT without IV contrast of the abdomen and pelvis demonstrated multifocal airspace disease and a left paraspinal abscess (Figure 1).

**Table 1:** Laboratory findings on admission Values are from the initial presentation. Arrows indicate abnormal values (↑ elevated, ↓ decreased). ^*^ Troponin reported as a high-sensitivity assay; reference range may vary by institution.

Parameter	Value	Unit	Reference range
Chemistry			
Calcium	8.5	mg/dL	8.6-10.2
Albumin	2.7 ↓	g/dL	3.5-5.0
Total protein	7.9	g/dL	6.0-8.3
Sodium	135 ↓	mmol/L	136-145
Potassium	4.7	mmol/L	3.5-5.0
Chloride	104	mmol/L	98-107
Bicarbonate (CO₂)	9 ↓	mmol/L	22-28
Blood urea nitrogen	140 ↑↑	mg/dL	7-20
Creatinine	13.2 ↑↑	mg/dL	0.6-1.3
Estimated glomerular filtration rate	4 ↓	mL/min/1.73 m²	>60
Glucose	153 ↑	mg/dL	70-100
Total bilirubin	0.8	mg/dL	0.1-1.2
Alkaline phosphatase	368 ↑	U/L	44-147
Aspartate aminotransferase	231 ↑	U/L	10-40
Alanine aminotransferase	196 ↑	U/L	7-56
Lactate	2.3 ↑	mmol/L	0.5-2.0
Cardiac marker			
Troponin	199 ↑	ng/L^*^	3-58.9
Hematology (CBC)			
White blood cell count	19.8 ↑	×10³/µL	4.0-11.0
Hemoglobin	10.7 ↓	g/dL	12.0-16.0
Platelet count	60 ↓	×10³/µL	150-400
Arterial blood gas			
pH	7.18 ↓	—	7.35-7.45

The patient was admitted to the intensive care unit with septic shock and was treated with IV fluid resuscitation and norepinephrine for hemodynamic support. Empiric antimicrobial therapy with linezolid and cefepime was initiated. Interventional radiology performed CT-guided drainage of a left paraspinal collection on hospital day 1, which yielded purulent material. Blood cultures and cultures of the drainage specimen both grew methicillin-sensitive *S. aureus *and extended-spectrum beta-lactamase *Escherichia coli*, prompting escalation to meropenem on hospital day 3. MRI of the lumbar spine without contrast showed persistent L4-L5 discitis-osteomyelitis with a possible anterior epidural abscess and high-grade spinal canal stenosis. The left internal jugular tunneled dialysis catheter was removed on hospital day 4 following positive blood culture results.

**Figure 2:**
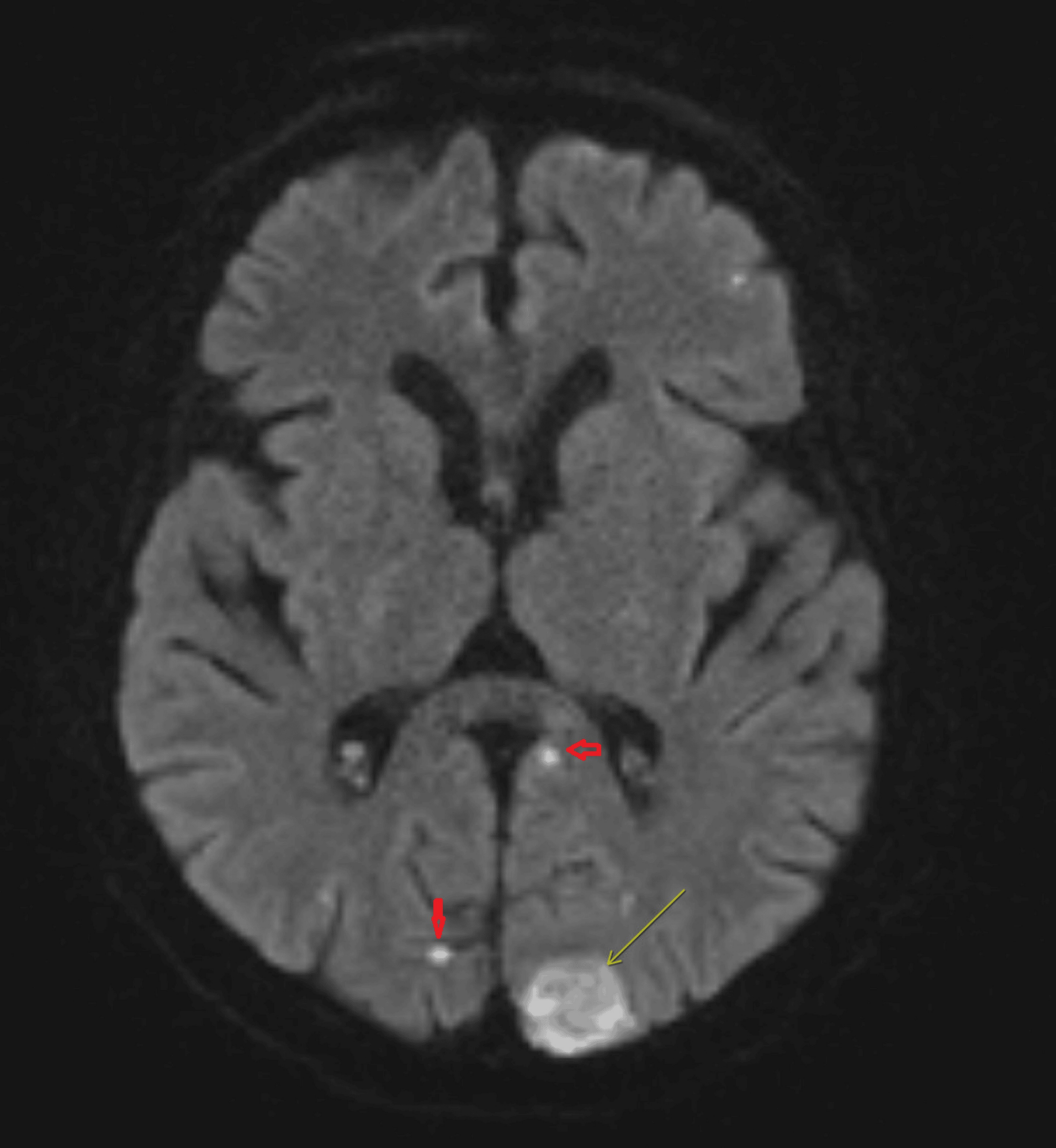
Diffusion-weighted imaging of the brain demonstrating multiple foci of restricted diffusion (red arrowhead and yellow arrow), appearing consistent with septic emboli Multifocal acute infarcts involving the medial right cerebellar hemisphere, left occipital lobe, and scattered bilateral frontoparietal and occipital subcortical white matter.

**Figure 3:**
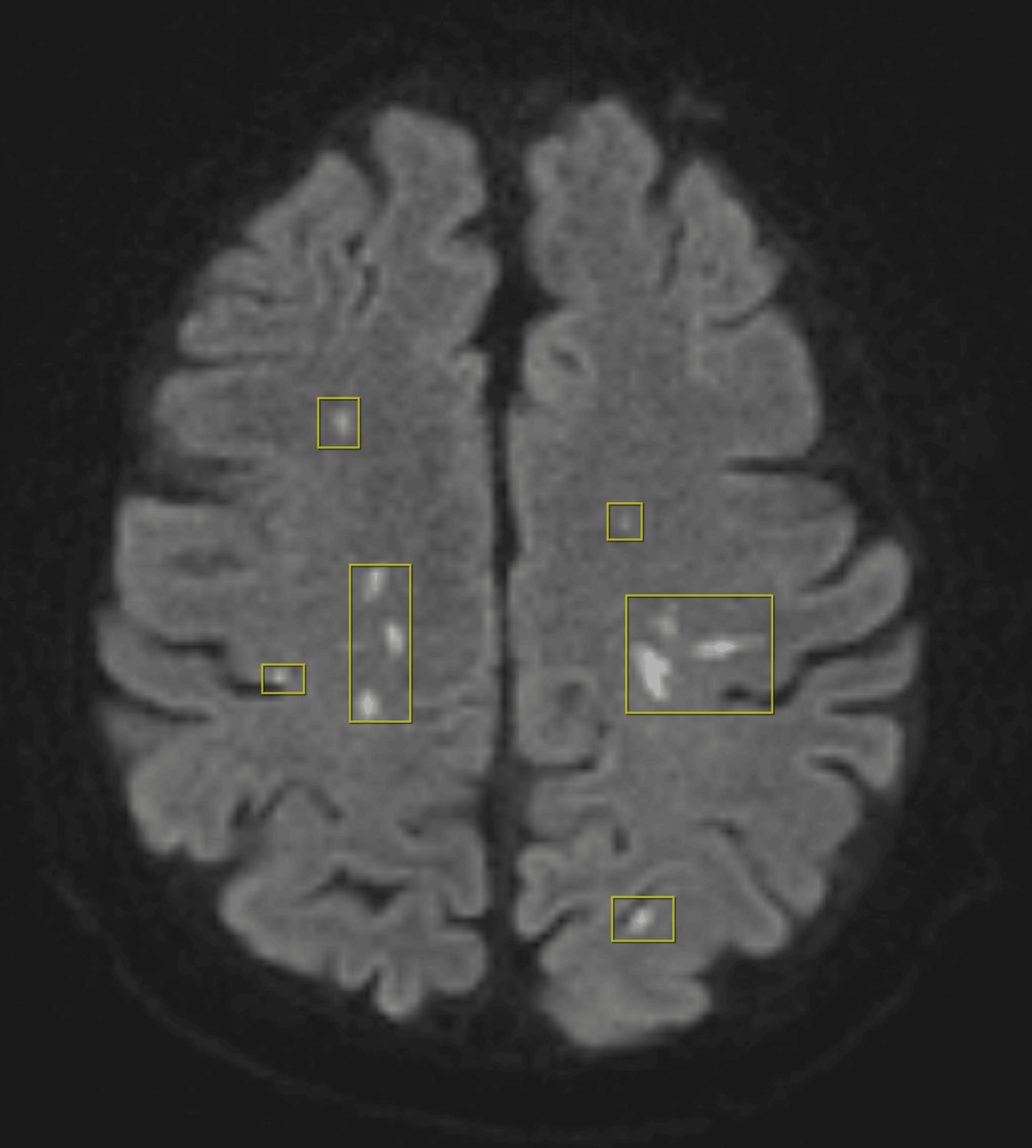
Diffusion-weighted imaging of the brain showing multiple areas of restricted diffusion in the cerebrum consistent with septic emboli (rectangle) Multifocal acute infarcts involving the medial right cerebellar hemisphere, left occipital lobe, and scattered bilateral frontoparietal and occipital subcortical white matter.

On hospital day 3, the patient also developed acute altered mental status with left gaze deviation. While awaiting transthoracic echocardiography, bedside point-of-care ultrasound (POCUS) demonstrated a mobile vegetation-like thickening of the posterior mitral valve leaflet (Video 1), highly suggestive of IE, which was later confirmed by transthoracic echocardiography showing a severely thickened posterior mitral valve leaflet with a mobile echodensity and mild-to-moderate mitral regurgitation. Based on persistent bacteremia and echocardiographic evidence of a new valvular vegetation, the diagnosis of IE was established by fulfillment of two major Duke criteria [13]. Same-day MRI of the brain revealed multifocal acute infarcts involving the right cerebellar hemisphere, the left occipital lobe, and bilateral frontoparietal subcortical white matter (Figure 2, Figure 3), consistent with embolic strokes. Magnetic resonance angiography of the head and neck showed no large vessel occlusion or hemodynamically significant stenosis.

**Video 1:** Parasternal long-axis POCUS demonstrating thickening of the posterior mitral valve leaflet, suspicious for vegetation POCUS, point-of-care ultrasound

Despite broad-spectrum antibiotics and percutaneous drainage, the patient’s leukocytosis persisted. Infectious disease consultants emphasized that definitive source control had not been achieved and strongly recommended transfer to a tertiary center capable of combined cardiothoracic and spine surgical intervention. His prognosis was considered poor without valve replacement and spinal infection control. Cardiothoracic surgery was consulted and recommended achieving definitive source control of the paraspinal abscess prior to consideration of mitral valve replacement. Neurosurgery assessed the patient and determined that operative intervention for the spinal infection carried substantial risk in the setting of severe thrombocytopenia, persistent hypotension, and overall medical instability. In light of the extensive ischemic stroke burden, profound functional decline, and multiple comorbidities, the patient was considered an unfavorable candidate for surgical intervention. As source control was not feasible and clinical status continued to deteriorate, prognosis was discussed with the next of kin. Following goals-of-care discussions, code status was changed to DNR/DNI, and care was transitioned to comfort-focused measures. The patient subsequently died following transfer to hospice.

## Discussion

This case illustrates the severe complications of IE in an ESRD patient on chronic hemodialysis with spinal hardware and a recurrent paraspinal abscess in the setting of persistent polymicrobial bacteremia. Compared to the general population, dialysis patients are more vulnerable to developing IE due to repeated vascular access, frequent exposure to intravascular devices, and immune dysfunction [1-5]. Dialysis patients who develop IE experience significantly increased mortality, with in-hospital mortality being about 30% and six-month mortality approaching 40%, and one-year mortality frequently exceeding 30-50% in published cases [1-3,5,7]. Persistent or recurrent bacteremia presents at a significantly higher rate in this group than in non-dialysis patients and often reflects challenges in achieving durable source control, especially in the presence of hardware or sustained infection [2,7,12].

Embolic events are among the most frequent and devastating complications of IE, occurring in roughly one quarter to one half of cases, with ischemic stroke reported in up to one-third of patients [6,8,9]. Risk is highest in the early phase of disease, particularly within the first two weeks after initiating antibiotics, reflecting active vegetation growth, inflammatory instability, and the lag before antimicrobial sterilization [14,15]. In addition, risk is strongly associated with mitral valve involvement (especially the anterior leaflet), large (>10 mm) or highly mobile vegetations, and virulent organisms such as *S. aureus* [9,14]. Notably, posterior leaflet vegetations can also embolize, as demonstrated in this case. Gram-negative and polymicrobial IE are further associated with higher mortality and narrowed antimicrobial options, particularly in patients with impaired renal function who cannot tolerate nephrotoxic agents or standard dosing strategies [7,10].

This case also illustrates the growing role of POCUS as an additional diagnostic tool in suspected IE. Transthoracic echocardiography and transesophageal echocardiography remain the definitive diagnostic modalities for IE. Transthoracic echocardiography is the recommended initial imaging study, with a sensitivity of 50-90% for detecting vegetations in native valve endocarditis [14,15]. When clinical suspicion remains high despite a negative or suboptimal transthoracic study, transesophageal echocardiography offers superior sensitivity (90-100%) and is preferred for ruling out IE [14,15]. POCUS serves as an extension of the physical examination, rapidly identifying findings that raise clinical suspicion and accelerating the pathway to definitive imaging or serving as a valuable bedside tool while awaiting formal echocardiography. Cardiac POCUS can rapidly detect valvular vegetations or significant regurgitation at the bedside, expediting diagnostic suspicion in unstable or atypical presentations [16]. Recent evaluations suggest that POCUS has a sensitivity of around 70-80% and a specificity of over 90% for detecting valvular vegetations, supporting its use to “rule in” IE when typical findings are visualized [16]. While POCUS cannot replace comprehensive transthoracic or transesophageal echocardiography, its use in high-risk septic patients with recurrent bacteremia or new neurologic deficits can expedite diagnosis and promote earlier multidisciplinary involvement.

Early surgical intervention is a cornerstone of guideline-directed management for complicated left-sided IE, particularly in cases involving heart failure, highly resistant organisms, persistent bacteremia or fever despite appropriate therapy, or recurrent embolic events with residual vegetations. Modern guidelines also recommend considering early surgery for patients with large, mobile vegetations greater than 10 mm, given their elevated embolic risk [8,14,17]. Dialysis-dependent patients with IE undergo cardiac surgery far less often than non-dialysis patients, despite observational data indicating that operative management is associated with improved long-term survival in this high-risk group. In published cohorts, only a small minority of hemodialysis patients receive surgical intervention (for example, 9.5% vs 51.7% in one series), and they experienced substantially higher in-hospital mortality and lower overall survival compared with non-hemodialysis patients [1,3,11]. In this case, extensive stroke burden, poor neurologic recovery, and multiple comorbidities ultimately precluded valve surgery and definitive spinal source control.

## Conclusions

Patients with ESRD on chronic hemodialysis are vulnerable to severe IE and embolic complications, especially when infection and hardware impede source control. In this case, recurrent polymicrobial bacteremia from a paraspinal source, in addition to mitral valve involvement, resulted in multifocal septic embolic strokes, illustrating how uncontrolled infection in dialysis-dependent patients can severely limit therapeutic options and worsen neurologic and overall outcomes. Clinicians should maintain a high index of suspicion for IE in hemodialysis patients with recurrent or unexplained bacteremia, particularly in the presence of spinal instrumentation or other prosthetic material. Bedside POCUS may serve as a useful adjunctive tool to raise early suspicion and facilitate timely triage; however, it should not replace comprehensive transthoracic or transesophageal echocardiography, which remains the diagnostic standard for confirming endocardial involvement and guiding management. Multidisciplinary collaboration among nephrology, infectious diseases, cardiology, cardiothoracic surgery, and spine surgery, along with timely referral to centers capable of advanced combined medical and surgical care, is essential to evaluate surgical candidacy and manage source control while a viable operative and treatment window still exists.
